# Interfacial Energetic Level Mapping and Nano-Ordering of Small Molecule/Fullerene Organic Solar Cells by Scanning Tunneling Microscopy and Spectroscopy

**DOI:** 10.3390/nano10030427

**Published:** 2020-02-28

**Authors:** Irving Caballero-Quintana, Daniel Romero-Borja, José-Luis Maldonado, Juan Nicasio-Collazo, Olivia Amargós-Reyes, Antonio Jiménez-González

**Affiliations:** 1 Research Group of Optical Properties of Materials (GPOM), Centro de Investigaciones en Óptica, A.P. 1-948, 37150 León, Gto., Mexico; icaballero@cio.mx (I.C.-Q.); romero_borja@ucsb.edu (D.R.-B.); nicasio.collazo@cio.mx (J.N.-C.); olivia@cio.mx (O.A.-R.); 2 Renewable Energies Institute (IER), Universidad Nacional Autónoma de México (UNAM), Priv. Xochicalco S/N, Mor. C.P. 62580 Temixco, Mexico; ajg@ier.unam.mx

**Keywords:** DRCN5T, thermal annealing, solvent vapor annealing, scanning tunneling microscopy, scanning tunneling spectroscopy, organic solar cells

## Abstract

Using scanning tunneling microscopy (STM) and spectroscopy (STS) at the liquid/solid interface, morphology evolution process and energetic level alignment of very thin solid films (thickness: <700 pm), of the low molecular weight molecule DRCN5T and DRCN5T:[70]PCBM blend are analyzed after applying thermal annealing at different temperatures. These films exhibit a worm-like pattern without thermal annealing (amorphous shape); however, after applying thermal annealing at 120 °C, the small molecule film domains crystallize verified by X-ray diffraction: structural geometry becomes a well-defined organized array. By using STS, the energy band diagrams of the semiconductor bulk heterojunction (blended film) at the donor-acceptor interface are determined; morphology and energy characteristics can be correlated with the organic solar cells (OSC) performance. When combining thermal treatment and solvent vapor annealing processes as described in previous literature by using other techniques, OSC devices based on DRCN5T show a very acceptable power conversion efficiency of 9.0%.

## 1. Introduction

In the last two decades, bulk heterojunction (BHJ) organic solar cells (OSCs) have received great attention due to their advantages, including low cost, flexibility, lightweight, and roll-to-roll processing compatibility [1,2]. To date, remarkable photon conversion efficiency (PCE) over 17% for polymeric ternary OSCs has recently been reported [3]. OSCs based on small molecules possess prominent advantages because the low molecular weight molecules, unlike polymers, have well-defined chemical structures, reduced batch-to-batch variability, fine tunable energy level and absorption, intrinsic monodispersity, etc. [4]. Solution-processed small-molecule based solar cells (SM-OSCs) have made great advances and a PCE over 14% has been achieved [5].

Among various approaches for device optimization, morphological control over the active layer plays an extremely important role in order to increase the photovoltaic characteristics of OSCs [6]. The BHJ photoactive layer provides energy offset between the donor and the acceptor to separate the excitons, therefore, morphology of the active layer in OSCs has strong influences on light absorption, exciton dissociation, charge transport and charge recombination; thus, it is of paramount importance for the overall photovoltaic performance [7]. As has been demonstrated, a key factor that limits the performance of OSCs is the phase-separation and domains size in the BHJ approach [8]. As a result, a series of treatment methodologies including incorporation of additives, thermal annealing (TA) and solvent vapor annealing (SVA) have been employed to control the morphology in the blend, and therefore, effectively to improve the performance in OSCs devices [9,10,11,12]. The post-treatments could improve the absorption due to the film morphology conformation; also, they can facilitate the phase separation and, crystallinity can be enhanced; these facts improve exciton-generation rate and charge mobility; also, there exist increased π-π stacking interactions between molecules of the same nature [7,13]. For instance, 2,2′-[(3,3′′′,3′′′′,4′-tetraoctyl[2,2′:5′,2′′:5′′,2′′′:5′′′,2′′′′-quinquethiophene]-5,5′′′′-diyl)bis[(Z)-methylidyne(3-ethyl-4-oxo-5,2-thiazolidinediylidene)]]bis-propanedinitrile (DRCN5T) small-molecule-based OSCs devices, after proper active layer post-treatments, can achieve PCEs up to 10% [10].

It is well known that small domain size is more efficient for exciton dissociation, however, not exactly for the charge transport issues. On the contrary, excessive phase separation or large domain size is favorable for charge transport but limits the exciton dissociation [14]. Thus, a proper balance between exciton dissociation and charge transport facts should be achieved in the BHJ approach. TA and SVA treated OSCs not only enhance light absorption but also improve charge dissociation and charge transport owing to the better nanoscale morphology. Therefore, it is necessary an optimized nanoscale phase separated morphology for the active layer in order to have efficient OSCs [14]. The phase-separated domains at the nanoscale regimen for DRCN5T small molecule are not yet investigated in detail, neither the thermodynamics nor kinetics morphology evolution processed by thermal treatments and SVA [8,15].

Some techniques, such as grazing-incidence wide-angle X-ray Scattering (GIWAXS), atomic force microscopy (AFM) and transmission electron microscopy (TEM) have been employed to clarify the OSCs performance improvement after applying TA or/and SVA treatments to their active layers [8,9,10,11,12]. However, in this work the film morphology evolution is analyzed at the nanoscale, the phase separation domains between donor and acceptor (DRCN5T:[70]PCBM as active layer) and the energetic band alignment at the donor-acceptor interface through scanning tunneling microscopy (STM) and scanning tunneling spectroscopy (STS) at the liquid/solid interface, when thermal annealing is provided to the small molecule DRCN5T as well as to the blend DRCN5T: [70]PCBM. Here, the very thin films (c.a. 700 pm) of DRCN5T and DRCN5T:[70]PCBM blend, were analyzed only for TA at different temperatures (room temperature = WO, 80, and 120 °C) by STM/STS because these very thin organic films (necessary fact required by these used techniques) are destroyed by applying the SVA process due to their thinness. STM/STS measurements provide the nano-morphology conformation and local density of states (LDOS) information of the organic samples, respectively [16,17]. Gaining knowledge over the morphology mechanisms and evolution in the blend, and correlating this information with energy levels between donor and acceptor compounds, it is essential to provide a clear pathway to further improve the overall device performance. TA in combination with SVA of the DRCN5T:[70]PCBM active layer, showed a very acceptable PCE of 9.0% in our fabricated OSCs.

## 2. Materials and Methods

### 2.1. Thin Films Fabrication for STM/STS Measurements

DRCN5T and DRCN5T:[70]PCBM both compounds purchased from 1-Materials (1-Material – Organic Nano Electronic, Quebec, Canada) and used as received were dissolved in chloroform with a concentration about 30 µg/mL and then deposited on highly oriented pyrolytic graphite (HOPG) by the drop casting method (thickness <700 pm). The solid thin films were thermally annealed on a hot plate at 80 °C and 120 °C for 10 min, a lot of care was taken for not destroying the films due to their thinness.

### 2.2. OSC Fabrication

**Figure 1:**
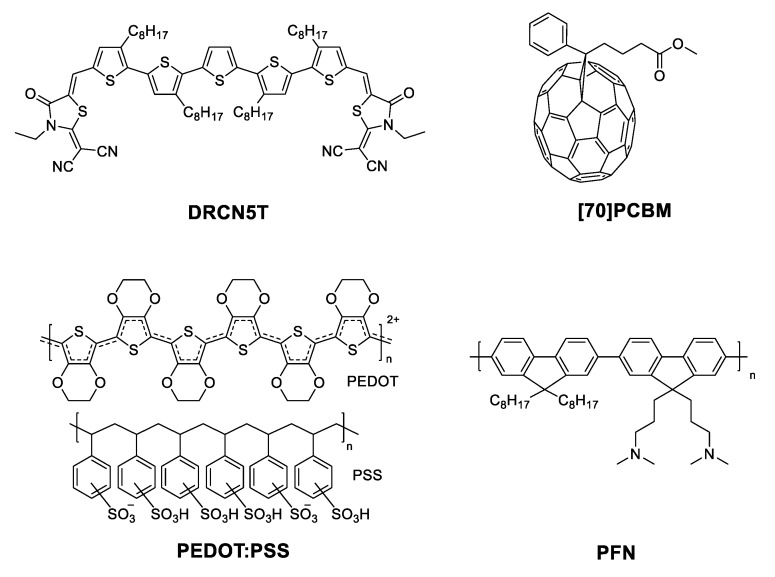
Chemical structures of the used materials in this work.

OSCs were fabricated under the BHJ approach in a direct configuration. ITO glass substrates (4–10 Ω/sq; Delta Technologies Ltd., Denver, USA) were cleaned sequentially with detergent; deionized water, acetone, and isopropyl alcohol in an ultrasonic bath for 10 min each, and then the substrates were treated with UV-ozone plasma for 20 min. First, a thin layer of PEDOT:PSS (thickness ~35 nm, Heraeus-Clevius P AI4083; Heraeus Holding, Hanau, Germany) was deposited in air conditions by spin-coating onto the pre-cleaned ITO, followed by a drying process at 120 °C during 30 min. DRCN5T:[70]PCBM active layers were spin-coated under N2 atmosphere in a glove box from chloroform solution (1:0.8 w/w, 18 mg/mL) achieving films with a thickness of 120 nm. After deposition, active layers were used with the following conditions: (a) without further post-treatment (WO), (b) thermally treated (TA) at 120 °C during 10 min on a hotplate under a controlled atmosphere of N2, (c) Solvent vapor annealing (SVA) treated (the samples were placed in the middle part of a Petri dish containing 120 µL of chloroform for 60 s, outside of the controlled N2 atmosphere) and (d) After applying thermal annealing (TA) at 120 °C, on a hot plate during 10 min, samples were cooled down to room temperature (all this procedure under a controlled N2 atmosphere) and then placed in a glass Petri dish (outside of the controlled N_2_ atmosphere) containing 120 μL of chloroform, 60 s of solvent vapor annealing (SVA) treatment was provided. Samples were then removed from the Petri dish and left on a plate at room temperature again for 10 min for proper drying of the thin films (remember that chloroform rapidly evaporates because its ebullition point is just 61 °C, further, SVA treatment is mainly for the sample surface). Finally, a thin film of PFN polyelectrolyte (spin-coated, thickness ~8 nm; Ossila Ltd., Sheffield, UK) as electron extraction layer (ETL) and Field’s metal (FM, drop-coated; RotoMetals, CA, USA) as an alternative top electrode, were deposited at regular atmosphere conditions as described in our previous works [17,18,19]; the active area was 0.04 cm^2^. The general OSC configuration was glass/ITO/PEDOT:PSS/DRCN5T:[70]PCBM/PFN/FM. Figure 1 shows the chemical structure of DRCN5T, [70]PCBM, PEDOT:PSS, and PFN.

### 2.3. Characterization and Measurements

STM/STS experiments were carried out with the Nanosurf Easyscan 2 STM/AFM (Nanosurf AG, Liestal, Switzerland) operating at constant current mode. STM/STS/AFM equipment is placed on a vibration isolation system (optical table) and, an electromagnetic shell covered the STM/AFM heads during measurements to avoid, as much as possible, noisy signals. For STM determinations, to decrease thermal drift, the atomic lattice of HOPG was scanned for approximately one hour. Mechanically cut platinum iridium (Pt-Ir) wires were used as STM tips. Before each film deposition, HOPG substrates were cleaved by using the adhesive tape technique to obtain an atomically clean surface; then, a droplet (about 2–5 μL) of 1-phenyloctane was directly deposited between the tip and the sample surface to make the liquid/solid interface for STM/STS measurements. The tunneling conditions of each STM image are given in the corresponding Figure caption. Images were processed and analyzed with Gwyddion open source software (v. 2.49). The photovoltaic parameters were extracted from J-V curves measured under AM 1.5 G illumination at 100 mW/cm^2^ by using a solar simulator class AAA (Sciencetech SS150; Sciencetech Inc., Ontario, Canada) calibrated with a Si reference cell (acquired from Abet technologies Inc, Milford, USA) and a Keithley 2450 (Tektronix Inc., Oregon, USA) source-meter unit.

## 3. Results and Discussion

**Figure 2:**
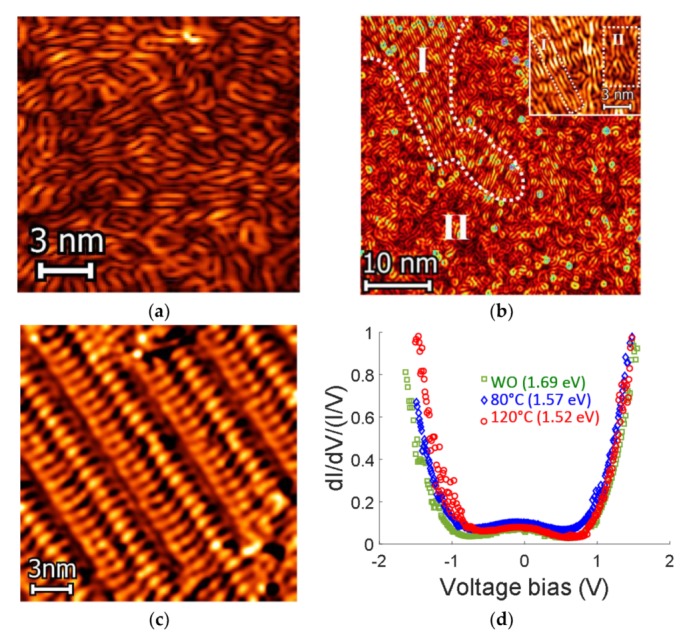
Scanning tunneling microscopy (STM) and spectroscopy (STS) of DRCN5T films analysis at different temperatures: (**a**) Without thermal annealing (WO), i.e., room temperature; tunneling conditions: It = 200 pA, U = 850 mV, (**b**) TA@80 °C, inset shows different reordering (structure I and structure II); tunneling conditions: It = 200 pA, U = 850 mV, (**c**) TA@120 °C; tunneling conditions: It = 200 pA, U = 950 mV, (**d**) Local density of states (LDOS) showing average electronic band gap variations (plot width correlation).

STM images and STS plots of reordered processed films, based on the small molecule DRCN5T, are displayed in Figure 2. The amorphous type structure was firstly measured after deposition of a drop from diluted solution (30 µg/mL) on HOPG substrate (by drop casting technique) at room temperature (without thermal annealing (WO)), showing a worm-like pattern (Figure 2a). In Figure 2b and its inset, after depositing the solution, the HOPG substrate was heated up at 80 °C/10 min. As it can be seen, STM image reveals two different assembly structures formed by DRCN5T (differentiated by the white dotted line), referred as structure I and structure II. Structure I shows a better ordering pattern (crystalline type, see also inset), meanwhile structure II remains as worm-like pattern (amorphous type). DRCN5T films show a complete reordering with a thermal annealing of 120 °C (Figure 2c).

**Figure 3:**
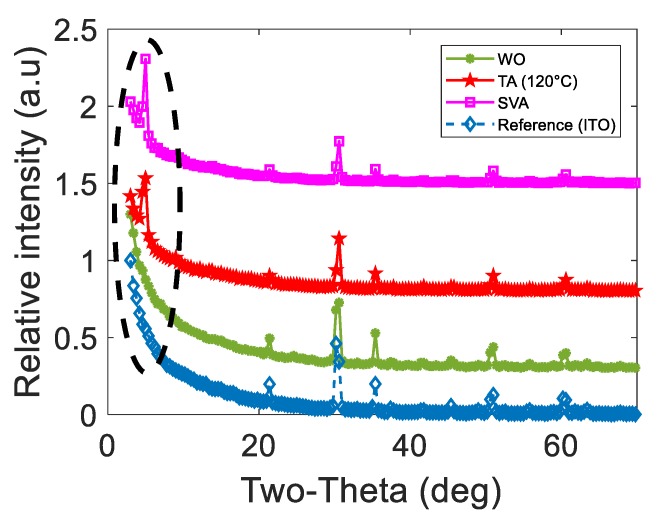
X-ray diffraction (XRD) pattern of DRCN5T films (Thickness ~160 nm, deposited onto ITO as the active layers for OSCs).

In Figure 3, at room temperature (WO), X-ray diffraction (XRD) does not show Bragg peaks (the peaks that are observed correspond to the reference substrate: ITO) confirming the amorphous behavior of the DRCN5T films (something similar was determined for DRCN5T:[70]PCBM films blend). On the other hand, after applying thermal (120 °C) or SVA (or TA + SVA) treatments to films, it can be seen a sharp Bragg peak around five two-theta degrees (indicated by a discontinuous black ellipse) due to a crystalline ordering, which is consistent with previous reports through 2D GIWAXS patterns [8].

This film reordering (structure I formation) can be attributed to the fact that when temperature increases, molecular translational and vibration motions could take place [20]. Increased temperature causes that molecular surface vibrates producing overall spinning motions, and therefore, the reordering [20]. It is worth noting that in 2D self-assembly systems, the intermolecular attractions between molecules and alkyn chains can generate a columnar packing pattern [21,22]. Additionally note that structure I from Figure 2b shows, to some extent, a brighter STM contrast (pointed out by the white dotted line; see also inset of Figure 2b) than that of structure II; according to the working principle of STM, a brighter STM contrast represents a higher conductance (because in Figure 2c there is a complete reordering, the contrast disappears) [22].

STM can visualize the nano-morphology conformation of DRCN5T films; further, the electronic properties and the local density of states (LDOS) at the Fermi energy (FE) can be determined from STS measurements [23]. Figure 2d shows the average local density of states at the given TA (WO, 80 and 120 °C). Typically, when a faster STS plot is recorded, it is better to eliminate instabilities or temperature-induced drift effects while running the spectroscopy experiments, however, the tunneling barrier height might oscillate, leading to a noisy plot [24,25]. To ensure reliable measurements of the estimated band gaps, the averaged results were derived from at least 15 experimental plots to achieve the differential conductance curve, which is proportional to the LDOS [24,25,26]. LDOS spectra were measured with a set point current tunneling (It) of 200 pA and a bias voltage of 850 mV with a modulation time of 300 ms for a better comparison by using a grid lattice (consisting of a matrix of 16 × 16 pixels, I–V curves were taken at each pixel) with a scan size of 15 × 15 nm (for TA = 80 °C, the scanning area correspond to the inset in Figure 2b).

**Figure A1:**
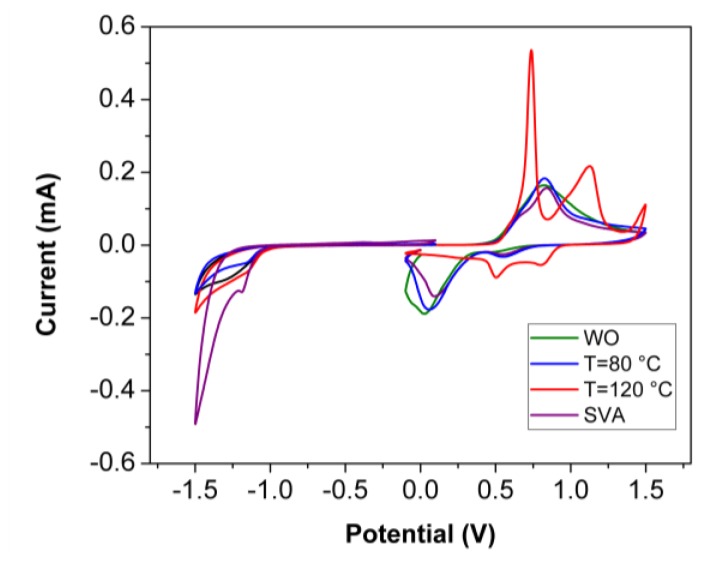
Cyclic voltammetry of DRCN5T films at different post-treatments.

From LDOS spectra (Figure 2d) it can be determined a bandgap reduction (width of the curve) between WO curve (1.69 eV), TA = 80 °C (1.57 eV) and TA = 120 °C (1.52 eV), probably due to the DRCNT5 film reordering, which result in a better interaction between each single molecule [26,27,28,29]. For the case of TA at 80 °C, the LDOS spectrum is consistent with a weaker (than for the TA = 120 °C case: complete reordering) electron coupling due to the different intermixed conformation structures I and II, and, stronger than for the WO case (amorphous type) [30]. Kan et al. [10] reported an optical band gap estimated from the onset of the DRCN5T film absorption of 1.60 eV, meanwhile the electrochemical band gap determined by cyclic voltammetry (CV) measurements was 1.81 eV [10], both of them without thermal treatment. Here, HOMO and LUMO energy levels were also determined by cyclic voltammetry (see Appendix A Figure A1), where variations in the band gaps are likewise observed for the DRCN5T films under different post-treatments. For WO the band gap (E_g_) was 1.51 eV, for TA@80 °C and TA@120 °C the values were 1.55 eV and 1.60 eV, respectively; and for SVA, E_g_ was 1.53 eV. These bandgap values are somewhat different from those determined by STM measurements, where a band gap reduction was measured when thermal treatment (at 120 °C) was applied to the DRCN5T film. This discrepancy could be due to the different used technique, the reference substrate and film preparation to determine these band gaps, for instance, for STM/STS measurements, film thickness was <700 pm (by drop casting), meanwhile for CV it was 120 nm. Table 1 summarizes the energetic levels HOMO and LUMO and bandgaps for the DRCN5T films at different post-treatments.

**Figure 4:**
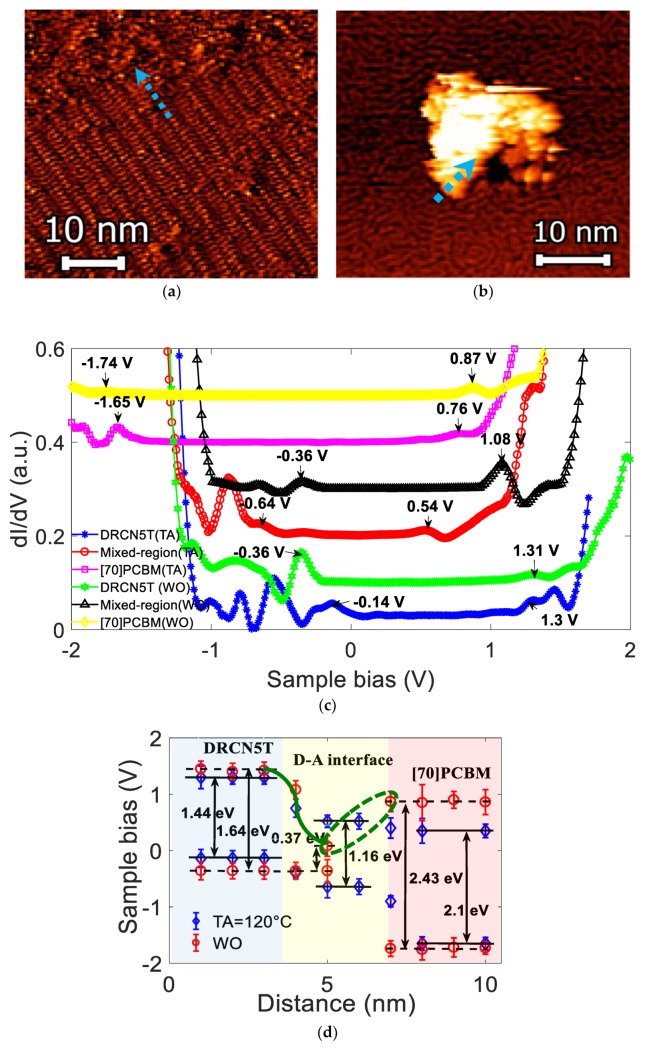
STM and STS of DRCN5T:[70]PCBM blend films, (**a**) Without thermal annealing (WO); tunneling conditions: It = 200 pA, U = 950 mV, (**b**) TA@120 °C; tunneling conditions: It = 200 pA, U = 950 mV, (**c**) Average LDOS measurements across the DRCN5T:[70]PCBM film domains, (**d**) Energetic band alignment across the DRCN5T:[70]PCBM domains.

The energetic values described in Figure 2d are only for the single DRCN5T molecule film at three different thermal annealing conditions. On the other hand, for the OSCs active film, the donor (D) compound is blended, under the bulk-heterojunction (BHJ) approach, with an acceptor (A); thus, Donor-Acceptor (D-A) nanoscale domains are formed. Figure 4a shows a STM image for binary blend (DRCN5T:[70]PCBM) thin film (deposited at room temperature). It shows a [70]PCBM-rich domain with a size of ~15 nm, however, it can also be observed the worm-like pattern of the small molecule DRCN5T film (see also Figure 2a). In Figure 4b, after applying a TA@120 °C, the blended film is reordered (see also Figure 2c). The good compatibility between D-A exhibits a smooth and uniform morphology after applying TA@120 °C.

Tskipuri et al. [31] studied the structure evolution in monolayers of PCBM via ultra-high vacuum scanning tunneling microscopy (UHV-STM), in which, molecular organization was monitored from disordered structures (aggregated clusters with a size of 10–25 nm, which is consistent with those of [70]PCBM observed in Figure 4a) to ordered arrangements driven by thermal annealing (from 20 °C to 250 °C) (in Figure 4b is no longer possible to detect [70]PCBM clusters at TA = 120 °C). Here is not showed any STM image at TA@80 °C since it was difficult to achieve stable images due to the combined presence of structures I and II (amorphous and crystalline shapes) of both the molecule DRCN5T and [70]PCBM at the same time. The blue dashed arrows on the STM images (Figure 4a,b) indicate the location along where the tunneling spectra were recorded in the donor/acceptor interface contact by using a linear path (point to point). Previous studies [16,32] reported that domains of the BHJ at the nanoscale could be identified because of the energy-level-mapping nature of the STS technique, allowing the possibility of drawing appropriate energy band diagrams. Furthermore, Cochrane et al. [33] studied the electronic density and energy level alignment of donor-acceptor interface with UHV STM/STS (samples held at ~4.4 K), which have significant implications for device design because level alignment strongly correlates to device performance [33]. Generally, donor and acceptor molecules form relatively pure phases, separated by a mixture of these compounds at the nanoscale level, which is called D-A interface [30,32].

Figure 4c shows the energetic difference across D-A interface with respect to TA conditions. In STS measurements, the zero sample bias indicates the Fermi level of the system [16]. A displacement to the left of the LDOS spectra indicates an electron-conducting (n-type) semiconductor, meanwhile a displacement to the right indicates hole conducting (p-type) semiconductor behavior, with the specific HOMO and LUMO levels located at negative and positive bias voltage, respectively [16,21,22]. By using a cross-sectional scanning tunneling microscope (XSTM) in UHV, Shih et al. [16] reported an E_g_ = 2.3 eV for PCBM (TA = 150 °C) with HOMO and LUMO levels located at −1.6 V and 0.7 V, respectively; from Figure 4c, the HOMO level is located at −1.65 V and the LUMO level at 0.76 V for PCBM (TA = 120 °C) leading to an average E_g_ = 2.1 eV, which is consistent with the literature reports [16,34]. Average energy levels from Figure 4c are plotted in Figure 4d. Energy level of the D-A interface tends to be different in comparison to pure phases due to the electronic density differences of the mixture [35], promoting either charge recombination or effectively leading to a good transport of the photogenerated and separated charges from the interface to the pure domains. Energy levels of the DRCN5T and [70]PCBM domains (Figure 4d) show a larger band gap for WO (1.64 eV for DRCN5T and 2.43 eV for [70]PCBM) in comparison with those of the domains on films TA treated (1.44 eV for DRCN5T and 2.1 eV for [70]PCBM). Note that in the case of the thin BHJ films without any post-treatment (Figure 4d, WO = open red circles) the energetic level LUMO from the DRCN5T to the D-A interface decreases (green arrow), and when it goes from D-A interface to the [70]PCBM acceptor, LUMO level of the D-A interface is smaller than the LUMO of [70]PCBM (open red circles), see inside of the dashed green ellipse. This difference between the energy levels of the D-A interface and [70]PCBM domains, could contribute to charge recombination and thus, reflected in a lower PCE value because the decreased charge collection at the electrodes. In contrast, the energy levels of the D-A interface with TA = 120 °C (open blue diamonds), show a better energy match, which could facilitate the charge transfer between D-A and be manifested as an improvement in the charge extraction [34,35] and, in an enhanced PV device performance.

Although previous reports [6,8] have analyzed the morphological control of the bulk heterojunction by using different post-processing strategies (TA, SVA and TA + SVA) and techniques (AFM, TEM, GIWAXS) of DRCN5T:[70]PCBM blend, to the best of our knowledge, no previous studies have been carried out in order to directly observe the morphology evolution process of DRCN5T molecule and DRCN5T:[70]PCBM blend, as well as the energetic level alignment at the D-A interface upon different annealing treatments; further, these studies were carried out by using STS and STM techniques; moreover, these measures were performed at the liquid/solid interface approach (room environmental conditions) [17,25], this approach is cheaper, easier and faster of performing than that of the UHV STM/STS systems (however, with less quality/resolution). All these stated reasons could help to better understand the PV performance in OSC devices.

**Figure 5:**
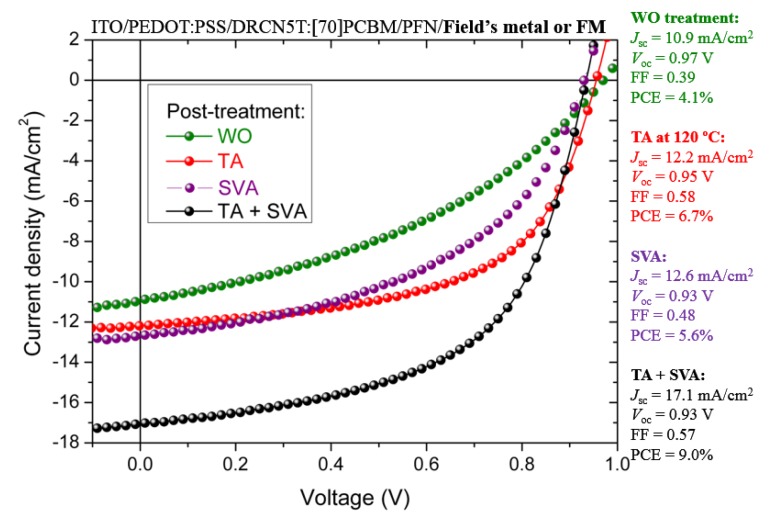
J-V characteristics of the fabricated OSCs without any treatment and with TA@120 °C, SVA with chloroform vapor and TA + SVA combination.

Figure 5 shows the J-V plots of OSCs based on DRCN5T:[70]PCBM. Fabricated devices with untreated (WO) active layers show a PCE of 4.1% with J_sc_ = 10.9 mA/cm^2^, V_oc_ = 0.97 V and FF = 0.39, while a great improvement in the photovoltaic performance is clearly observed once thermal annealing at 120 °C was given: PCE = 6.7 %, J_sc_ = 12.2 mA/cm^2^, V_oc_ = 0.95 V and FF = 0.58. For just SVA treatment, PCE was 5.6% with J_sc_ = 12.6 mA/cm^2^, V_oc_ = 0.93 V and FF = 0.48. The best PV performance of the fabricated devices under TA + SVA combination was PCE = 9.0% with J_sc_ = 17.1 mA/cm^2^, V_oc_ = 0.93 V and FF = 0.57, which is consistent with previous literature [6,8,10]. Kan et al. [10] reported an excellent thermal stability up to 360 °C of DRCN5T molecule under N_2_ atmosphere and an optimized TA@120 °C/10 min + SVA for DRCN5T:[70]PCBM active layer with a PCE = 10.1% [10]. In [6] the maximum reported PCE (TA + SVA) is 8.3%.

**Figure 6:**
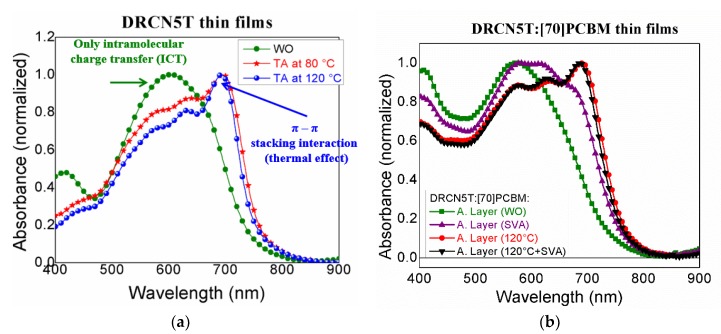
Absorption spectra of (**a**) DRCN5T thin films, and (**b**) DRCN5T:[70]PCBM thin films under different TA (and SVA for the case of the blend) treatments.

The efficiency improvement under TA + SVA combination is mainly due to the higher current output (FF also increases particularly under the TA treatment): It could be attributed to two facts: due to an increased absorption of photons inside the photoactive layer when TA is provided (see Figure 6b), which could improve the charge collection [36]. Likewise, when implemented the SVA treatment, an optimized contact between the active layer and the ETL could lead to an enhancement in the parallel (shunt) resistance, limiting the electrical losses (Jsc leakage) [8,37]. Then, an overall better PV performance when the active layer is TA + SVA treated can be achieved. Therefore, the mentioned post-treatments induce better morphological organization and corresponding local electronic properties to provide an impressive increase of the charge generation, transport/collection and thus, an enhanced device performance (up to 9.0%) of the SM-OSCs. As previously stated, because the very thin films (c.a. 700 pm) fabricated for the STM/STS analysis are destroyed by applying the SVA treatment due to their thinness, DRCN5T and DRCN5T:[70]PCBM blend were analyzed only for TA at different temperatures (WO, 80, and 120 °C). Usually, in OSCs the active layer thickness is about 100 nm. For these thicker films, other techniques (such as SEM, X-ray, etc.) should be used to analyze their conformation and/or possible quasi-crystalline array. Table 2 shows comparisons of photovoltaic performance with previous literature (at different post-treatments).

As shown in Table 2, under the different TA treatments and SVA process, Voc value slightly decreases—(different for the Jsc value case and in less extend for the FF value), which may be within the experimental error. It is well known that the donor HOMO and acceptor LUMO difference correlates with Voc value, then, according to results discussed from Figure 4c that partially explain the Jsc enhancement (and in less extent also FF) a Voc variation should also be expected (actually, a Voc decrease); thus, further and deeper analysis should be carried out in this sense. However, Voc value depends on multiple factors as mentioned in [38,39].

Figure 6a shows the optical absorbance of DRCN5T thin films where, after two different thermal annealing processes (80 °C and 120 °C/10 min), a peak at 693 nm appears due to the π-stacking between DRCN5T molecules compared to the untreated thin films (WO). In the case of thin films treated at 120 °C, the peak at 693 nm has a steeper slope than for the film treated at 80 °C due to the well-ordered pattern (see Figure 2a–c). With respect to the active layer comprised of DRCN5T:[70]PCBM (Figure 6b), it is also observed similar variations for thin films without treatment (WO) and for TA@120 °C; additionally, here is also showed the blended film absorption when SVA and TA(120 °C) + SVA were provided.

**Figure 7:**
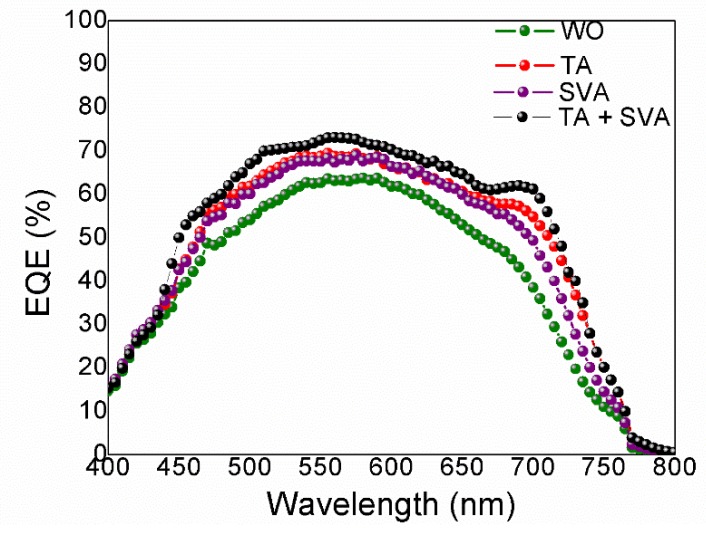
EQE spectra of OSCs devices based on DRCN5T:[70]PCBM films with different treatments. Thermally treated (TA) at 120 °C during 10 min and SVA with chloroform vapor for 60 s.

The absorbance for thin films treated at 120 °C and 120 °C + SVA is fairly the same (and without the pronounced peak at 693 nm just for SVA). Probably, the effect of SVA is the improvement of molecular ordering mainly at the surface level rather than in the whole film bulk, facilitating a better interface contact with the electron transport layer (ETL) [8]. From AFM measurements, the active layer surface exhibit, without thermal treatment, a root-mean-square (RMS) roughness (R_q_) of 0.85 nm. While under TA, SVA and TA + SVA treatments, roughness is 1 nm, 0.68 nm and 0.47 nm, respectively. In Figure 7, external quantum efficiency (EQE) [40] measurements are shown; for the active films thermal treated, it can be seen a shoulder between 675 and 750 nm with a maximum peak near to 693 nm (that correlates with the observed absorption in Figure 6b) as commented above due to the π-π stacking interactions between DRCN5T molecules.

## 4. Conclusions

In this work, through STM/STS (at the liquid/solid interface approach), thin film analysis and energetic band alignment of the DRCN5T:[70]PCBM domains, at different post-treatments, suggests that without thermal annealing, there is not good alignment of energy levels due to a disordered arrangements between D-A constituents, which could contribute to charge recombination decreasing charge collection at the electrodes. On the other hand, a good phase separation between donor and acceptor driven by a thermal annealing, leads to an energy level match and provides a better charge-collecting pathways, which is necessary to improve the performance of SM-OSCs. Thermal annealing results in a better nanoscale ordering of the small molecule DRCN5T films and also for the DRCN5T:[70]PCBM blend. TA effectively improves the photovoltaic performance of DRCN5T:[70]PCBM based SM-OSCs. Furthermore, when combining TA and SVA, PV performance improves even more: the reached PCE value was of 9.0%. The better ordering, after applying TA + SVA, provides a better electrical charge transport and collection, thus, improving the PCE value. Although STM and STS techniques, at the liquid/solid interface approach (room environmental conditions), have less quality/resolution than UHV STM/STS systems, they have the advantages of being cheaper, easier and faster of performing. The presented analyses could provide new insights for better understanding the fabrication and improved overall OSCs performance.

## Figures and Tables

**Table 1:** Energy levels and band gaps of DRCN5T small molecule at different post-treatments measured by cyclic voltammetry (CV, thickness = c.a. 120 nm onto ITO) and by scanning tunneling spectroscopy (STS, thickness = c.a. 700 pm onto HOPG).

DRCN5T Post-Treatment	E_ox, onset_ (eV)	HOMO (eV)	E_red, onset_ (eV)	LUMO (eV)	E_g_ (eV) by CV	E_g_ (eV) by STS
WO	0.49	−5.09	−1.02	−3.58	1.51	1.69
TA80	0.51	−5.11	−1.04	−3.56	1.55	1.57
TA120	0.52	−5.12	−1.08	−3.52	1.60	1.52
SVA	0.50	−5.10	−1.03	−3.57	1.53	-

**Table 2:** Comparison of the best PV parameters for DRCN5T:[70]PCBM BHJ based OSCs.

Reference	Thermal Treatment	*V*_oc_ (V)	*J*_sc_ (mA/cm^2^)	FF	PCE_best_ (PCE_av_) ^a^ (%)
[6]	WO	0.98	7.5	0.48	3.6
	TA	0.93	12.3	0.58	6.7
	SVA	0.95	12.6	0.55	6.6
	TA + SVA	0.93	14.4	0.66	8.3
[10]	TA + SVA	0.92	15.7	0.68	10.1
This work	WO	0.97	10.9	0.39	4.1 (3.4)
	TA	0.95	12.2	0.58	6.7 (6.1)
	SVA	0.93	12.6	0.48	5.6 (5.6)
	TA + SVA	0.93	17.0	0.57	9.0 (8.6)

^a^ The average PCE value was achieved from at least 3 devices.
